# The American Medical Association

**Published:** 1881-07

**Authors:** 


### Medical and Surgical Journal.

VOL. XIX.] JULY—1881. [No. 4

### Reports of Societies.

### THE AMERICAN MEDICAL ASSOCIATION.

The thirty-second annual meeting of the National Association was held in Richmond, Virginia, commencing Tuesday, May 3, and continuing until Friday, May 6, 1881. Nearly five hundred delegates and members were in attendance.

According to the programme, the meeting was convened at eleven o'clock A.M., with prayer by Bishop J. J. Keane, after which Hon. F. W. M. Holliday, Govenor of the State, delivered an eloquent address of welcome.

The Committee of Arrangements announced the order of business, as well as the entertainment and invitations from several sources.

The President of the Association, Dr. John T. Hodgen, of St. Louis, then delivered his annual address, in which he referred to the two types of surgeon,—the bold, reckless operator, and the conservative, timid one,—and warned his colleagues against the temptation to perform brilliant operations when the patient's best interests would be furthered by a more tentative course. On the other hand, he urged early interference in proper cases, such as malignant disease, sarcoma of choroid, rodent ulcer, and sympathetic ophthalmia. Various conditions of the body tend to render an operation unsuccessful: previous vigorous health in itself will prevent a patient from patiently bearing confinement ; a strumous constitution, long-continued mental strain, anæmia, and wasting disorders, all tend to reduce the powers of recovery. Imperfect knowledge of many pathological states also would prevent the resort to the knife, in the absence of exact information of the state of the system. After referring to the many advances in operative surgery, he concluded by saying that a more perfect animal chemistry, a more thorough histology, and a deeper search into the possibilities of pathological change, will doubtless throw many a ray of light into regions where the darkness is now too dense for our vision to penetrate. To these fields coming generations of physicians will surely be attracted, in the faith that, as man advances in knowledge and approaches somewhat nearer to the comprehension of the perfect wisdom which designed the wonderful physical organism through which he is brought into relation with the world around him, he will be enabled to solve more and more of the difficult problems which now perplex and baffle us, and will gradually raise medicine to a position more nearly akin to that now accorded to the exacter sciences.

The address was attentively listened to and warmly applauded. A vote of thanks was passed to Dr. Hogden, and the paper referred to the Publication Committee.

Dr. Jos. H. Warren, of Boston, chairman of Foreign Delegates appointed at the last meeting, reported attendance upon the British Medical Association at Cambridge, and gave an interesting report of the proceedings at that meeting. In conclusion, he spoke of the *British Medical Journal*, and advocated the establishment of a similar official periodical by the Association.

PROCEEDINGS IN THE SECTIONS.

*Section I.—On the Practice of Medicine, Materia Medica and Physiology.*—Dr. Wm. Pepper, Philadelphia, Chairman ; Dr. T. A. Ashby, Baltimore, Secretary.

In the absence of the chairman, Dr. Octerloney, of Louisville, occupied the chair.

Dr. W. C. Wile, of Connecticut, read a paper on "Blood-

Letting as a Therapeutic Measure in Pneumonia," in which he advocated the free use of the lancet in the early stage, and reported several cases apparently greatly relieved by the venesection.

Dr. J. J. Lynch, of Baltimore, protested against this method of treatment, as the disease runs its course in spite of the bleeding, and the patients recover just as soon without it. When bleeding was common, the mortality of pneumonia was about twenty-five per cent.; now it is scarcely more than four to five per cent. Dr. Whittaker, of Cincinnati, also entered a vigorous protest against this proceedure, which he considered as being unjustifiable. Veratrum viride, also recommended by the lecturer, is a profound cardiac sedative, and might do much harm.

Dr. N. S. Davis advocated the treatment, and Dr. S. D. Gross said that it was applicable to the first stage in young, robust subjects, where he considered it to be the great remedy. The subject was still further discussed until the time of adjournment.

*Section II.—On Surgery and Anatomy.*—Dr. Hunter McGuire, Virginia, Chairman; Dr. Duncan Eve, Tennessee, Secretary.

Dr. Jos. H. Warren, of Boston, exhibited a number of instruments, among which were catheters and bougies with a screw or vermicular point; a modification of Thompson's evacuating tube for the bladder after crushing a stone, or for removing foreign bodies; a uterine probe with a rotary point, a trocar, and a set of sounds for the urethra or uterus on the same principle. Also a serrated herniotome, torsion forceps, a hernia syringe, and a peculiar dissecting-knife·

Drs. Sayre and Gouley, of New York, could not discover anything in the revolving point·to commend its use.

Dr. Wm. A. Byrd, of Quincy, Ill., reported a case of ulceration of the appendix vermiformis, with remarks upon abdominal section in cases of perforation of the bowel. In the discussion, operative interference was strongly recommended.

Dr. James E. Reeves, of West Virginia, exhibited a renal calculus, weighing 480 grains, from a case of pyonephrosis. A number of smaller calculi coexisted, weighing in the aggregate 416 grains.

*Section III.—Obstetrics and Diseases of Women.*—Dr. James R. Chadwick, Boston, Chairman; Dr. Joseph Tabor Johnson, Washington, Secretary.

Dr. Paul F. Munde opened the discussion upon vaginal and vagino-abdominal pessaries, and formulated a number of propositions as guides to their use, as follows:

1. Always be sure of the diagnosis, of the nature and degree of the displacement, before resorting to a pessary.

2. Always replace the uterus before applying a pessary, especially in retro-displacements. Preliminary treatment by frequent replacement and straightening the canal with a sound will often be beneficial.

3. Never insert a pessary when there is evidence, by the touch, of acute or recent inflammation of the uterus or adnexa, or when pressure by the finger on the parametrium (where the pessary is to rest) gives decided pain.

4. When the uterus is not replaceable,—that is, when adhesions bind the fundus down,—use great caution and discrimination in deciding whether an attempt shou'd be made and is justified by the symptoms to elevate the fundus by manual or instrumental means, or whether the elevation should first be tried by the gradual elevation of a pessary (this applies only to retro- and latero-versions). If neither is to be recommended, do not introduce a pessary until local alterative and absorbant measures have effected a resolution of the adhesions.

5. Always choose an indestructible instrument, if possible. This does not apply to prolapsus uteri.

6. Always measure and estimate the vagina carefully before choosing a pessary, and be careful to adjust the pessary in every particular (size, curve, width) to that particular case. No two vaginæ are exactly alike.

7. If the vaginal pouch is not sufficiently deep to accommodate a pessary (anterior pouch for ante-displacements), defer the attempt to fit a pessary until the pouch has been deepened by daily tamponing with cotton, or by the upward pressure of a Cutter or a Thomas vagino-abdominal supporter. Or the pouch may be gradually deepened by using first a small (slightly curved in retro-displacement) instrument and gradually increasing its size (or curve) until the desired size and shape for permanency are reached.

8. Never leave a pessary in the vagina which puts the vaginal walls to the stretch, and which does not permit the passage of a finger between it and the wall of the vagina. This does not apply to prolapsus uteri.

9. A vaginal pessary which projects from the vulva is displaced.

10. A pessary which gives pain must at once be replaced by one which is painless.

11. A well-fitting, properly-chosen pessary should not only give no pain, but should be a direct source of comfort to the patient.

12. Always examine a patient on her feet after introducing a pessary, or when it is desired, at her return, to ascertain its efficiency in sustaining the uterus during walking and exertion.

13. Always tell the patient that she has a pessary in her vagina, or she may not return in spite of your directions, and the pessary may remain for years, to her ultimate great discomfort and danger.

14. Always tell the patient to return within a week after the first introduction, in order that the position and working of the pessary may be looked after, and that, if it does not suit, it can be removed and a better one inserted. Tell her that several trials and various instruments may be required before one is found which she can wear permanently. Also let her return for inspection once every four to eight weeks, as the case may require. Tell her that if she fails to do so the pessary may cause ulceration, for whicn treatment will be needed.

15. Tell the patient that she will need to wear the pessary for months, perhaps years, before a recovery can be expected.

16. Never introduce a pessary which the patient cannot remove herself.

17. Tell the patient to remove the pessary herself if it gives her pain, and show her how to do it. When she has removed it, let her present herself at once for examination.

18. Tell the patient to use daily vaginal injections for cleaning purposes. If she notices profuse discharge, add astringents; if the discharge is sanious or purulent, let her come at once, as the pessary has probably caused abrasion.

19. Tell her on moving the pessary to test the result; that the permanence of the result cannot be determined for several days or weeks.

20. Always direct your patient to relieve the superincumbent pressure by a proper support of their skirts. If the displacement be anterior, aid the pessary by an abdominal supra-pubic pad.

All pessaries may be introduced in the knee-chest position when it is desirable or possible to replace the uterus only in that position. A Sims speculum elevates the perineum, the air enters and expands the vagina, and the pessary (chiefly in retroversion and prolapsus) is introduced by touch and sight, and the patient laid over on her left side. For aggravated retroversion and for prolapsus of ovaries or uterus, this position offers many advantages over the semi-prone decubitus. Care must be taken to remember that the position of the patient is reversed, and that the pessary must be introduced accordingly.

Dr. R. Beverly Cole spoke in favor of the Hodge pessary and his own modification of it with a spring support for retroversion. He also advocated the use of the galvanic stem pessary in the condition of atony. In conclusion, he exhibited a gas cautery for operations upon fungous growths, etc, and demonstrated the method of moulding gutta-percha, previously softened in warm water, so as to make extemporaneous pessaries.

Dr. Albert H. Smith deprecated the indiscriminate use of pessaries; he did not believe in the galvanic action of the stem pessary mentioned, but considered its effects merely mechanical.

A number of other speakers united in discouraging the use of stem pessaries.

*Section V.—On Ophthalmology, Otology, and Laryngology.*—Dr. Dudley S. Reynolds, Kentucky, Chairman; Dr. S. M. Burnett, District of Columbia, Secretary.

Dr. W. C. Jarvis, of New York, read a paper on "Nasal Catarrh with Hypertrophy," and advocated transfixion of the hypertrophied portion by curved needles, and its removal by wire ecraseur in preference to the galvano cautery or the knife. The paper was followed by a general discussion of nasal catarrh and its causes.

Dr. Chisolm, of Baltimore, read a paper on "Conical Cornea," in which a case was reported benefitted by tapping with a red-hot needle.

Dr. Little, of Philadelphia, also spoke of a case improved after tapping.

*Section VI.—On Diseases of Children.*—Dr. A. Jacobi of New York, Chairman; Dr. T. M. Roach, Boston, Secretary.

A paper was presented by Dr. H. J. Bowditch, of Boston, on "The Relation between Growth and Disease," in which the rate of development was mentioned, as throwing light upon the causation and treatment of children's diseases. He advocated frequent weighing of children, a progressive loss of weight indicating the approach of some sickness. The subject is to be considered by the National Board of Health, which will distribute blank cards for statistical information, some of which were exhibited by the lecturer.

Dr. S. C. Busey, of Washington, presented a paper by Dr. White, of Boston, "On some of the Causes of Infantile Eczema, and the Importance of Mechanical Restraint in its Treatment."

Dr. L. R. Bulkley thought that the author had laid too great stress upon the local treatment, and had ignored hygienic and other means. He approved of the disuse of water, but had never been obliged to resort to the use of the mask.

Dr. Busey read a paper on "The Relation of Meteorological Conditions to Diarrhœal Diseases of Children."

Dr. H. O. Marcy exhibited some elastic double canula injection tubes for irrigation of stomach, rectum, bladder, vagina, etc.

Dr. W. H. Goodwillie, of New York, read a paper, illustrated by means of a model, on the method of preventing thumb sucking. The treatment recommended consists in breaking up the habit by applying a leather pad to the elbow, preventing the hand from coming to the mouth.

Co-existing nasal catarrh should be treated by douches and the application of powder blown into the nose, proper food, clothing and rest.

His conclusions were as follows:

(1.) Thumb-sucking is more disastrous to the health of the child than the sucking of the other fingers, for, the thumb once in the mouth, it more readily remains during sleep.

(2.) It interferes with the child's proper rest, which should be continuous and undisturbed, and so become a source of nervous irritation and exhaustion.

(3.) It interferes with the natural respiration through the nose, and sets up abnormal conditions.

(4.) It malforms the anterior part of the mouth, and affects future mastication.

SECOND DAY'S PROCEEDINGS.

The amendment to the Code of Ethics, which at the last meeting was made the order of the day for this session, was brought before the Association by the President. The amendment (to Article I., Paragraph 1st) read as follows: "and hence it is considered derogatory to the interests of the public and the honor of the profession for any physician or teacher to aid in the medical teaching or graduation of persons knowing them to be supporters and intended practitioners of some irregular and exclusive system of medicine.

After a motion to table the amendment had been voted down, the subject was laid over until the next morning.

*Publication of Proceedings.*—Dr. John H. Packard, chairman of the committee to whom the recommendations of the late President's annual address were referred, made an interesting report, concluding with the following resolution:

"*Resolved*, That the President be authorized to appoint a committee of five to digest and report in detail as soon as practicable upon the time, place, and terms of the publication of such a journal, to elect an editor, fix his salary, and to arrange all other necessary details."

After some discussion, Dr. Davis moved to strike out so much of the resolution as related to the election of an editor. Adopted. On motion, the secretary and treasurer were added to the committee.

Dr. Toner offered a resolution instructing the secretary to publish, with the forthcoming report of the Transactions of the Association, the index of the proceedings of all the previous meetings. Adopted.

Dr. William Pepper, chairman of the Section on Medicine, read an able address, discussing recent theories of disease, and suggested that many conditions now considered as typical of blood-poisoning might be due, as originally taught by Broussais, to some internal morbid condition, such as inflammation of mucous membranes, chiefly catarrhal in character.

The chairman of the Section on Obstetrics, Dr. Chadwick, of Boston, read his address upon the Advance of Obstetrics and Gynæcology during the past five years, which contained a valuable resume of interesting cases, important operations, and statistics of publications in this department of practical medicine.

Both addresses were referred to the Committee of Publication.

PROCEEDINGS IN THE SECTIONS.

*Section I.—Practice of Medicine, Materia Medica and Physiology.*—Dr. Wm. Pepper, Philadelphia, Chairman ; Dr. T. A. Ashby, Baltimore, Secretary.

By appointment, Dr. King read a paper by Dr. Prentiss, of New York, entitled "Is Croupous Pneumonia a Zymotic Disease?" after which Dr. Dabney read a paper on "The Nature and Treatment of Pneumonia," which elicited an animated and prolonged discussion, without any general agreement, upon the subject of the etiology of pneumonia.

Dr. Robinson, of New York, being absent, his paper was read by title, "On the Nature and Treatment of Pulmonary Phthisis," and referred to the Publication Committee.

Dr. L. Duncan Bulkley, of New York, presented a paper on the "Diet and Hygiene of Eczema," which was deferred until the next afternoon.

*Section II.—Surgery and Anatomg.*—Dr. Hunter McGuire, Chairman; Dr. Duncan Eve, Secretary.

Dr. Charles F. Stillman, of New Jersey, read a paper on a "New System of Surgical Mechanics," and presented a number of splints. He summed up the advantages of his system as follows: 1. Extension at any angle with motion. 2. Extension at any angle with luxation. 3. Fixation. 4. Motion complete or limited, constant or occasional. 5. Exposure of surface about the joint, admitting compression, elastic or otherwise; hot and cold applications; blisters; dressings; and easy inspections.

Dr. Alfred C. Post, of New York, read a paper on "Plastic Operations on the Face," and reported two cases. The first case which he related was one of epithelioma, involving the left side of the face, about fifty-five millimetres in diameter, extending above to a line within six millimetres of the margin of the lower lid, and below to a line midway tween the ala of the nose and the angle of the mouth. The patient was a man 61 years of age. Dr. Post operated by making a horizontal incision separating the morbid growth from the lower lid, and another parallel incision below the tumor, connecting the two by vertical incisions before and behind. He then dissected up from the adjacent parts the diseased mass included between the four incisions, leaving a quadrilateral chasm, nearly of a square form. The two vertical incisions were then extended downward, the anterior one to a little below the base of the lower jaw, and the posterior one nearly as low as the base of the jaw. From the inferior extremities of these two vertical incisions, curved incisions, with their concavities looking backward and upward, were made to the extent of five centimetres, including between them a peduncle of skin and subjacent tissues for the nourishment of the extensive flap. The flap was then drawn up and secured by sutures so as to fill up the whole space left vacant by the removal of the disease. The wound healed throughout, leaving the patient but slightly disfigured.

The second case was one of absence of the upper lip and of the columna nasi, as well as of a portion of the right ala nasi, occasioned by the application of a caustic paste for the removal of a supposed cancer of the upper lip. The patient was a man 65 years of age. Dr. Post operated on the 1st of May, 1880. He commenced by separating from the cheek a small remnant of the upper lip, which remained at the left extremity, and reversing it so as to form a columna for the nose. He then made two horizontal flaps from the cheeks with peduncles curved downward and forward, and united them by means of pins and sutures, so as to reconstruct the upper lip. A number of supplementary operations were required, and the result was a marked improvement in the appearance of the patient.

Water-color drawings were exhibited, showing the conditions of these two patients before and after the operations, which showed the benefit and added much to the interest of the paper.

Dr. D. H. Goodwillie, of New York City, read a paper on "Treatment of Arthritis of the Temporo-Maxillary Articulation," in which he reported cases treated. The causes of arthritis are local and constitutional. Whenever it occurs, the motion of the jaws becomes impaired according to the cause, severity and length of the disease, and they often require long treatment in order to restore the muscles to their normal condition.

The treatment is by means of apparatus to relieve the joint of pressure on the inflamed articular surfaces. It is made as follows. An impression of the teeth of the entire jaw is taken, and an interdental splint made, the posterior part of which is thickened a little, for the purpose of a fulcrum.

Another impression is taken of the chin, and a rubber splint is made to fit it. A skull-cap is next made to fit the head closely, with elastic bands on each side passing down from it and fastening to the chin-splint.

The interdental splint is placed in position in the mouth, and the back teeth of the jaw closed on the fulcrum of the interdental splint; then, when pressure is made on the chin by tightening the elastic bands connecting the skull-cap with the chin-splint, the joint is relieved from pressure.

Dr. Moore, of Rochester, called attention to cases where cicatricial bands caused the trouble--division of which usually cured them.

Dr. S. D. Gross, of Philadelphia, had seen comparatively few such cases since the abuse of calomel had ceased. He had not been able to accomplish much with wedges. Alluding to section of the bone, he said he had several times performed the operation.

Dr. Hunter McGuire, of Richmond, Virginia, said that one of the cases alluded to by Dr. Goodwillie afterwards fell into his hands. The case was not cured. There was no motion whatever. Dr. McGuire took out a weged-shaped piece of bone just at the angle, and the result was good. He also spoke of a case of eleven years' standing, where a bridge of bone passed from the upper to the lower jaw, treated in a similar manner by excision of the bridge of bone.

Dr. A. L. Sayre exhibited the mode of applying a plaster jacket to a case of lateral curvature.

On motion of Prof. Gross, a vote of thanks was passed to Dr. Sayre. In discussing the method, Dr. Hodgen, of St. Louis, criticised the jury-mast, and said that it affected the growth of the jaw, which Dr. Sayre said he had never observed. Dr. Hutchison, of New York, spoke in favor of his method of treatment of hip-disease over that by the wire breeches.

The Chair appointed Drs. Kinloch, of South Carolina, Pratt, of St. Louis, and Burgett, of New York, as members of the Prize Award Committee.

*Section III.—Obstetrics and Diseases of Women.*—Dr. J. R. Chadwick, Boston, Chairman; Dr. J. T. Johnson, D.C., Secretary.

A prolonged discussion was held upon the nature of the investment of uterine fibroids. Dr. H. P. C. Wilson, of Baltimore, then exhibited his uterine dilator and other instruments, which gave an opportunity for a protest from several members against instrumental interference with the uterus upon two slight grounds, betraying a reaction against the common resort to dangerous procedures upon trivial excuses.

Dr. H. O. Marcy exhibited his double drainage and injection tubes, by invitation from the Chair.

*Section IV.—On State Medicine, etc.*—Dr. J. S. Billings, Chairman ; Dr. R. J. Jennings, Secretary,

Prof. J. L. Cabell read a paper on "The National Board of Health and the International Sanitary Conference of 1881." After a historical sketch of the labors of the National Board of Health and of the Conference which met in Washington last January, Dr. Cabell coucluded as follows :

"There is, therefore, good reason for hoping that an international agreement may be arrived at between the States most frequently threatened with epidemic invasions. Aside from this, the degree of attention which, as a result of the deliberations of the conference, has been given to the subject of maritine sanitary police, cannot be without fruit in securing greater cleanliness, better ventilation of ships sailing on the high seas, and, in general, an improved sanitary condition of these important instruments of commerce, which become so often the carriers of the most deadly contagion, from the failure to use such precautions as sanitary science suggests, and as it is hoped will now be enforced by the maritime powers of the world."

Dr. C. F. Folsom, of Boston, read a paper on "The Relation of the State to the Insane," in which he compared the present methods of the treatment of the insane with those formerly in vogue. He also gave valuable statistical information as to the number of insane in various sections of the country ; and he argued in favor of the establishment of State Lunacy Boards. Among the points made were, first, a Lunacy Board should embrace men with a thorough knowledge of insanity and its treatment. The chief duties of this Board should be to secure proper care for those unfortunate insane who are kept in private dwellings, where they are very much liable to neglect. Secondly, they should require the commitment of lunatics to the asylum, and otherwise look into the cases, so as to be able to decide whether the lunatics should be retained for care or be discharged.

The paper was in every respect an able and thorough one, and full of information.

In discussing the paper, Dr. Grissom made the point that if insanity is treated properly it is comparatively curable; but the usual custom is to delay action too long. One reason of this is that some people seem to think it disreputable to have it known that they have an insane person in the family. When insanity becomes generally recognized as a purely physical disease and is so treated, a different feeling in regard to it will prevail, and a better prognosis will be afforded.

*Section V.—Ophthalmology, Otology, and Laryngology.*—Dr. D. S. Reynolds, Kentucky, Chairman; Dr. S. M. Burnett, Washington, Secretary.

Dr. Carl Seiler, of Philadelphia, read a paper on "Syphilitic Laryngitis," in which he discussed the diagnosis and treatment. He stated that the affection could be diagnosticated from non-specific inflammation by the peculiar discoloration of the mucous membrane and the symmetrical disposition of the inflammatory patches. There are frequent ulcerations of the larnyx, which may be divided into shallow ulcers (in nothing different from those seen in catarrhal laryngitis), and deep ulcerations, which were, in the author's opinion, due to the breaking down of the smaller or larger gummata in the mucous membrane. He also stated that a diagnostic sign of syphilitic laryngitis was seen in the red lines, and surrounding areola best observed upon the velum palati. He recommended as treatment (besides the systematic, with iodide of potassium and mercury, and supportive, with tonics, etc.), local touching of the shallow ulcers with solid nitrate of silver fused upon an aluminium probe, and the deep ulcers with acid nitrate of mercury (1 to 4), or the galvanic cautery.

In the discussion, Dr. Reynolds said that he thought constitutional treatment would do more good than any local measures. He was surprised at the statement of Dr. Seiler, that the diagnosis of simple form syphilitic laryngitis was difficult. He did not believe that catarrhal inflammation could produce ulceration. There were three kinds of syphilitic ulcers, viz.:

1. The ulcer with inflamed base and serrated edges.
2. Ulcer with pale or gray base, with irregular margin and sharply defined scarlet areola.

3. Ulcer wholly unaccompanied with any evidence of inflammation, with sharply defined outlines, and gummatous deposits surrounding the margin.

He thought that local applications could give no good results, unless restricted to such remedies as would give relief from pain.

The subject was also discussed by Drs. Stephens and Walsh.

Dr. Chisolm, of Baltimore, read a communication on a form of Tinnitus Aurium, due to rhythmical construction of the tensor tympani, coming on at intervals in the day, resembling the fluttering of the eyelid. Treatment was unsatisfactory. In one case the omission of wine at dinner gave permanent relief.

Dr. Eugene Smith, of Detroit, reported a case of blepharoplasty, in which transplanting of skin was performed (one and a half by two inches was the size of the portion taken from the arm).

Dr. H. Augustus Wilson, of Philadelphia, presented some compressed pellets of atropia for ophthalmic use. One containing one one-hundredth of a grain was sufficient to relax the accommodation in an hour.

Just before adjournment, a paper by Dr. Laurence Turnbull, of Philadelphia, entitled (1) Otitis Intermittens, with Observations upon the Use of Quinine in Diseases of the Ear; (2) On the Importance of Ear Examinations in Effecting Life Insurance, was, on motion, read by title, and referred to the Committee on Publication.

*Section VI.—Diseases of Children.*—Dr. A. Jacobi, New York, Chairman; Dr. T. M. Rotch, Boston, Secretary.

Dr. Jacobi made some introductory remarks upon eczema in infants, and advocated the use of restraint to prevent scratching. No water should be used, except in very rare contingencies. Scabs should be removed by alkaline lotions of Hebra's soap, to be followed by astringents, such as zinc ointment or diachylon. Attention to diet is of great importance, and often the children thrive better on artificial food than on breast-milk.

Dr. Jacobi, in the absence of Dr. R. J. Nunn, of Savannah, read a paper from him on the Treatment of Diphtheria. The disease had raged in Savannah with very fatal effects, and a letter from a friend elsewhere said that, after treating six hundred or seven hundred cases, his faith in the efficacy of drugs was very feeble. The causative influences are probably not the same in all cases. Medicines which cure the disease in Germany fail in this country; and the discussions as to the identity of croup, diphtheria, and scarlet fever are strong argument in favor of this belief, and all treatment based upon one cause must fail to relieve all cases. Dr. Nunn quoted Dr. Jacobi as saying, "The entrance of the diphtheritic poison into the system is not the same in all cases. There are cases in which the origin of the disease is decidedly local. There are others in which the poisoning of the blood through inhalation is the first step in the development of the disease." A powder highly recommended and used is as follows:

| | | |
|---|---|---|
| R.—Sulphur. sub, | gr. xlviij. | |
| Acid. tannic., | gr. xij. | |
| Acid. salicylic., | gr. i. | |
| Pulv. potass. chlor., | gr. xij. | M. |

Precaution must be used in compounding this prescription. A little of this powder is put on the back of the tongue every hour or two, and a small piece of ice given afterwards. It will be seen that this prescription is a combination of antiseptics principally.

In another case the following formula was used with good effect:

| | | |
|---|---|---|
| R.—Sulphur, | gr. vij. | |
| Acid. boracic, | gr. iv. | |
| Acid. tannic, | gr. j. | |
| Acid. salicylic, | gr. j. | |
| Resorcin, | gr. j. | M. |

Another formula is:

| | | |
|---|---|---|
| R.—Sulphur. sub., | gr. viij. | |
| Acid. boracic, | gr. iv. | |
| Acid. benzoic, | gr. j. | |
| Acid. salicylic, | gr. j. | |
| Acid. tannic, | gr. j. | |
| Acid. tartaric, | gr. iv. | |
| Sodii chlorid, | gr. iv. | |
| Resorcin, | gr. j. | M. |

Dr. Lathrop, of New Hampshire, said that he had experimented with chloroform largely, and finds it a highly useful agent. He uses it in diphtheria and other throat affections, applied on a piece of cotton attached to a tube or pen holder. The patients usually required visiting no longer than four days; but the cases were not so malignant as had been reported in other localities.

No unpleasant effects have ever followed this plan of treatment and the child, in true diphtheria, does not complain of *smarting* from the application of chloroform. He had used this plan of treatment in one hundred cases. Of course constitutional measures are added.

Dr. Wm. Lee, of Baltimore, has used equal parts of tinct. ferri chlorid. and ol. ricini with benefit. He considers the disease as local at first, and then constitutional. A physician from his county had used large doses of ol. copaibæ with benefit, followed by emetics to remove the membrane.

G. Vivian, of Minnesota, has used in severe epidemics alcohol as an inhalation, and has employed as much as a quart of alcohol a day. He has never seen any constitutional effects ensue.

Dr J. McNeal, of Gettysburg, Pa., recommends the following: Potass. bromid., ʒj; potass. chlorat., ʒij; acid. carbolic, gr. xx; aquæ, Oj. Use in an inhaler. Locally, chloroformi, ʒij; lin. saponis, ℥j–ʒij.

Dr. F. E. Hitchcock, of Rockland, Maine, uses equal parts of sulphurous acid and water in an atomizer. The proportions can be varied, and the acid used as a gargle, with cold affusion externally.

Dr. Jacobi, in answer to an inquiry concerning the benefit of pilocarpin, said that his opinion of it was unfavorable. It was proposed as a specific by a Dr. Guttman; and, while on this subject, he would state that this gentleman was not Dr. Guttman, of Berlin. His article on pilocarpin had struck him as nothing more than an advertising arrangement. In diphtheria there are two forms, one in which the deposit can be readily separated from the mucous surface beneath, and one in which the deposit is deeply imbedded in the lower structures. In the latter form Dr. Jacobi believes that pilocarpin does harm, and in one case he thinks that death was hastened by using this drug. Pilocarpin debilitates the heart's action, often giving rise to nausea and vomiting. The milder cases of diphtheria recover, as a rule, if left alone; and in all cases of the disease he thinks the utility of pilocarpin doubtful. The paper in which pilocarpin was recommended as a specific is not satisfactory as regards the description of the cases treated. If the remedy be used at all, a fluid extract is the best preparation, as the muriate of pilocarpin is decomposed in the stomach. His opinion also of the effect of lime was not so high as that of many writers.

Dr. E. H. Bradford presented a paper entitled "Resection of the Tarsus in Severe Cases of Club-Foot." The first case was that of a girl eleven years old, with severe equino-varus, the axis of the foot being at right angles with that of the leg. Tenotomy and mechanical treatment were tried for a month, with but slight benefit. Dr. Bradford removed a wedge-shaped section of bone from the tarsus with a metacarpel saw, and with antiseptic precautions. There were no constitutional symptoms after the operation (except that the temperature rose once to 101°, being otherwise normal during the recovery), and in five weeks the patient was able to walk without a cane. The second case was one of a boy thirteen years old, with club foot, in which an equally good result was obtained after operation.

THIRD DAY'S PROCEEDINGS.

After the announcement of the names of additional delegates, Dr. Hunter S. McGuire, of Virginia, as chairman of the Section on Surgery, read an address upon "Operative Interference in Gunshot Wounds of the Peritoneum," urging surgical interference, free drainage, etc.

Dr. John S. Billings, by vote of the Section on State Medicine, read the paper presented yesterday to the Section "On Some of the Results of the Tenth Census, as regards Mortality Statistics."

Dr N. S. Davis submitted a report on "Clinical Observations and Records." The report was accompanied by a recommendation that a committee of five be appointed by the President of the Association, to be called the Standing Committee on Atmospheric Conditions and their Relation to the Prevalence of Disease; also suggesting how the committee shall be appointed, etc., and that five hundred dollars be appropriated for expenses. The recommendations were adopted.

*Officers for* 1881.—Upon a question of privilege, Dr. Sayre, of New York, requested that the minutes of the session of 1877 be so amended as to contain his protest against a resolution adopted in the Surgical Section in regard to shortening after fracture of long bones, which, he explained, was sent to the secretary of the Association, and not to a Chicago newspaper, as had been publicly asserted. The amendment was ordered to be made, in accordance with the explanation.

Dr. Stone submitted the report of the Committee on Nominations, as follows: For President, J. J. Woodward, U. S. A., of Washington; First Vice-President, I. P. O. Hooper, of Arkansas; Second Vice-President, Leartus Conner, of Michigan; Third Vice-President, Eugene Grissom, of North Carolina; Fourth Vice-President, Hunter McGuire, of Richmond; Secretary, William B. Atkinson, of Pennsylvania; Treasurer, R. J. Dunglison, of Pennsylvania; Librarian, William Lee, of Washington, D. C.; Chairman of the Committee on Arrangements, Dr. Stone.

Vacancies in the Judicial Council were filled by the appointment of Dr. S. N. Benham, of Pennsylvania; Dr. J. M. Jones, of the District of Columbia; D. A. Linthicum, of Arkansas; William Brodie, of Michigan; H. D. Hollon, of Vermont; A. B. Sloan, of Missouri, and R. Beverly Cole, of California.

St. Paul, Minnesota, was selected as the next place of meeting.

On motion of Prof. Gross, the rules were suspended. He then offered a resolution to establish a Section on Dentistry, which, as an amendment, was duly laid over until next meeting.

The amendment to the Code of Ethics was then taken for discussion: "and hence it is considered derogatory to the interests of the public and honor of the profession for any physician or teacher to aid in any way the medical teaching or graduation of persons knowing them to be supporters and intended practitioners of some irregular and exclusive system of medicine."

Dr. Davis made an earnest speech in support of the amendment. He was followed by Dr. M. P. Martin, of Massachusetts, also in support of the amendment.

A motion to lay the subject on the table was lost—ayes 106, noes 108. The matter was again postponed, and made the special order for the next morning, immediately after the meeting of the Association.

The following amendment to the by-laws, offered by Dr. J. M. Keller, of Arkansas, then came up and was adopted :

" In the election of officers and appointment of committees of this Association by the President, he shall be confined to members in actual attendance, except in the Committee on Arrangements."

PROCEEDINGS IN THE SECTIONS.

*Section I.—Practice of Medicine.*—Dr. L. D. Bulkley finished his paper on "The Diet and Hygiene of Eczema," which he regarded as of great importance in treatment.

Dr. Whittaker, of Cincinnati, read a communication on the Treatment of Diphtheria, in which he recommended the use of antiseptics, such as quinine, salicylic acid, and the benzoates, and the local application of the officinal solution of the subsulphate of iron.

Dr. J. A. Octerlony presented a paper on the Treatment of Dysentery, which was referred.

Dr. I. E. Atkinson read a paper on " The Production of Albuminuria by the Use of Iodide of Potassium in Syphilitic Disease," which led to an interesting discussion.

Dr. H. Augustus Wilson showed his hypodermic pellets.

Dr. Henry A. Martin read a paper on Variola Vaccina and Variola Equinæ in Massachusetts, in which he reported a spontaneous case of *variola equinæ.*

In conclusion, he moved that this Section recommend to the Association that a committee be appointed to visit these various farms, and investigate the whole subject of bovine virus. Carried.

"The Materia Medica of the Future" was the title of a paper read by Dr. T. E. Stewart, of New York, and referred.

The following resolution, offered by Dr. Dunster, was adopted:

"*Resolved*, That the spirit of the Code of Ethics forbids a physician from prescribing a remedy controlled by a patent, copyright, or trade-mark. This, however, shall except a patent upon a process of manufacture or machinery, provided said patent be not used to prevent legitimate competition; and shall also except use of a trade-mark used to designate a brand of manufacture; provided that the article so marked be accompanied by working formulæ, duly sworn to, and also by a technical, scientific name under which any one can compete in the manufacture of the same."

Dr. Upshur, of Richmond, Virginia, read the report of a case of paralysis of motion of both upper extremities, in which the prominent symptoms were complete paralysis of both upper extremities *except in the hands*, intense pain in the shoulders on any attempt to lie down, and inability to sleep. The attack was ushered in by convulsions, epileptic in character.

*Section II.—Surgerg and Anatomy.*—Dr. Hunter McGuire, Chairman; Dr. Duncan Eve, Secretary.

Dr. B. A. Watson, of Jersey City, read an essay entitled "An Experimental and Clinical Inquiry into the Etiology and Distinctive Peculiarities of Traumatic Fever." After an historical resume, he proceeded to define "traumatic fever," which he regarded as a term often used improperly for "septic fever." Modern investigations lead us to admit the existence of two varieties of fever after wounds, a septic and a non-septic variety. In a series of physiological experiments upon rabbits, in which he succeeded in lowering the temperature by injections of ether, chloroform, or carbolic acid, in most cases absorption of the fluid and swelling took place without a rise of temperature; in the exceptional case abscess with septic poisoning, and death, resulted. After these careful experiments, the writer is convinced that the fever was not due to the lesion, and raised the question as to whether or not it is due to the absorption of carbolic acid. The carefully-made experiments of Edelberg apparently demonstrated that the traumal fever noticed in connection with the antiseptic treatment should be attributed to the partly congealed and partly fluid blood rather than to the carbolic acid. After reviewing the question thoroughly, Dr. Watson is confident that primary "antiseptic" fever is due to carbolic acid acting on the wound-secretion, aided by the air; whilst absorption always precedes true septic phenomena.

The paper was discussed by Drs. Campbell, Nancrede, Post, Quimby, and others.

Dr. Charles A. Leale read a paper on "Malignant Pustule, or Labial Carbuncle," for which he advocated free incisions and thorough cauterizing the cut surfaces with nitric acid, subsequently treating it as an open wound with balsam of Peru.

On motion, a committee of three was appointed to select six subjects for discussion in the Section on Surgery at the next session of the Association.

*Section III.—Obstetrics and Diseases of Women.*—The Chair announced the following committees:

Committee for Selection of Subject of Essay.—Prof. E. S. Dunster, Michigan; Prof. G. M. B. Maughs, St. Louis; Dr. H. M. Field, Boston.

Committee of Award.—Dr. Robert Battey, Georgia; Dr. Albert H. Smith, Philadelphia, Pa.; Dr. P. F. Munde, New York City.

Committee to which Papers for Publication were Referred.—Dr. Joseph Tabor Johnson, Washington, D. C.; Dr. John Byrne, Brooklyn, N. Y.; Dr. H. P. C. Wilson, Baltimore, Md.

Dr. Joseph Tabor Johnson read a clinical inquiry entitled "Can we make a positive diagnosis of pregnancy previous to the occurrence of the audible sounds of the fœtal heart and the detection of the fœtal movements?" After citing the presumptive evidences of pregnancy, he concluded that the increased temperature of the vagina afforded a means of positive diagnosis of early pregnancy.

The paper was generally discussed.

Dr. Robert Battey, of Georgia, was surprised that no gentleman had mentioned any of the following aphorisms in doubtful cases:

1st. Always consider a married woman pregnant, if living with her husband, until *proved* otherwise.

2d. Always consider an unmarried woman innocent till *proved* guilty.

3d. Always believe that a woman married, of the highest character, living with a husband of equally high character, both solemnly assuring the medical man that no intercourse has taken place for two years, as she has been bedridden for that length of time, may bring forth a dead fœtus.

4th. Always believe a young unmarried woman with abdominable tumor, of high social position and unimpeachable virtue, if she has been watched over by a platonic and abstemious young cousin of the male persuasion while the mother went out—to be pregnant.

Dr. P. F. Munde, of New York, exhibited some instruments of original device, after which a discussion was entered into upon the management of the third stage of labor.

*Section IV.—Medical Jurisprudence and State Medicine.*

No papers were read before this Section; two were referred by title to the Publication Committee—on "Cellars," by Dr. A. N. Bell, of Garden City, and on the "Necessity and Means of Removing Putrescible Matters from Inhabited Places," by Dr. W. Wright, of Milwaukee.

*Section V.—Ophthalmology, Otology and Laryngology.*

The afternoon was devoted to the discussion of Astigmatism, by Drs. Reynolds, Risley, Burnett, Scott and Daly.

A paper presented by the Chairman on the treatment of nasal polpi was read by title and referred.

*Section VI.—Diseases of Children.*

Dr. Blake, of Massachusetts, presented a paper on "Middle-Ear Disease in Children in the Course of the Acute Exanthemata."

Dr. Jacobi presented his address as Chairman of the Section, in which special attention was given to the exanthemata. Rubeola (German measles) he considered fully entitled to a position among the contagious constitutional affections. In diphtheria he protested against the use of large doses of chlorate of potassium, as being dangerous, many fatal cases being on record. The paper was followed by general discussion.

Dr. Rotch, of Boston, said that, in order that there should be no confusion of terms, it should be first stated that there was a true measles and a rubeola (the rotheln of the German writers), as stated by Dr. Jacobi, and that so far as the eruption was concerned it was often impossible to make a distinction; secondly, that roseola, which was usually a papular eczema, had nothing to do with the other diseases, and that in the great majority of cases the eruption caused by eating crabs was that of uticaria, which, of course, was easily distinguished from the eruption of measles; thirdly, that he would again state that undoubted cases of true rubeola had been observed in Boston, where the disease is well recognized and differentiated from measles.

FOURTH DAY'S PROCEEDINGS.

The President announced the following Committee on Clinical Observations and Records: Drs. N. S. Davis, Chicago; J. M. Toner, D. C.; H. A. Marcy, Boston; W. H Giddings, N. C.; S. M. Bemis, New Orleans.

*The Ethical Amendment.*—Dr. Billings, U. S. A., of Washington, submitted the following as a substitute for the amendment to the Code of Ethics which was under discussion yesterday: "It is not in accordance with the interests of the public, or the honor of the profession, that any physician or medical teacher should examine, or sign diplomas or certificates of proficiency for, or otherwise be specially concerned with the graduation of, persons who they have good reason to believe intend to support and practice any exclusive and irregular system of medicine."

Dr. Billings, in offering the paper, said, "This amendment has been prepared after consultation with the representatives of the various views which have been presented here, and it is believed to embody the prevailing opinion of the Association. It places no restriction upon teaching or upon the diffusion of knowledge, while it affirms the principle that we will not endorse in any way or recommend to the public men who limit their practice to one special dogma, or who deliberately class themselves with the vilifiers of and sneerers at regular medicine for the sake of notoriety."

The substitute, under the call of the previous question, was adopted.

The Committee on Nominations made their final report, naming the presidents and secretaries of the various Sections.

*Abuse of Medical Charities.*—The committee to which was referred the communication from the Philadelphia County Medical Society in reference to the abuse of medical charities reported the proposed plan of co-operation between the medical charities and Philadelphia Society for Organizing Charities to be admirably adapted, if faithfully carried out, to remedy the evil at which it is aimed, and which has reached such colossal proportions in that as in every large city both in this country and in Great Britain. As Philadelphia is exceptionally favored in having its systems of charity so admirably methodized and co-ordinated, your committee would suggest that in other cities not possessed of this machinery a concerted action of all the medical charities in combination with the police authorities, in the matter of the visitation and registration of the dependent classes, might be attended with valuable results. The report concluded with the following resolution:

"*Resolved*, That the secretary be instructed to convey to the officers of the Philadelphia County Medical Society the acknowledgement of the indebtedness of this Association to the Society for thus bringing to our notice its action in a matter so nearly concerning the dignity of the profession and the welfare of the public."

The resolution offered yesterday by Dr. E. Dunster in the Section on Practice, relating to patent medicines, was referred to the Judicial Council.

The report of the treasurer showed a balance of two thousand and eight dollars and forty-six cents in his hands.

The librarian, Dr. Lee, presented an interesting report, and asked that two hundred dollars be appropriated for binding and purchase of books, and that the treasurer be authorized to subscribe fifty dollars to the *Index Medicus*, both of which were adopted.

On motion, one thousand dollars were appropriated from the treasury for the permanent secretary for his services during last year.

*Report of Nominating Committee*—The Committee on Nominations reported the following additional officers, who were elected,

I. *Section on Practice of Medicine.*—Chairman, Dr. J. A. Octerlony, Kentucky; Secretary, Dr. Deering J. Roberts, Tennessee.

II. *Section on Surgery and Anatomy.*—Chairman, Dr. J. C. Hughes, Iowa; Secretary, Dr. William A. Byrd, Illinois.

III. *Section on Obstetrics.*—Chairman, Dr. H. O. Marcy, Massachusetts; Secretary, Dr. C. V. Mottram Kansas.

IV. *Section on Medical Jurisprudence and State Medicine.*—Chairman, Dr. A. L. Gihon, Washington, D. C.; Secretary, Dr. J. H. Sears, Texas.

V. *Ophthalmology, Otology, and Laryngology.*—Chairman, Dr. D. B. St. John Roosa, New York; Secretary, Dr. J. Solis Cohen, Philadelphia, Pa.

VI. *Diseases of Children.*—Chairman, Dr. S. C. Busey, Washington, D. C.; Secretary, Dr. William Lee, Baltimore, Maryland.

VII. *Dentistry.*—Chairman, Dr. D. H. Goodwillie, New York; Secretary, Dr. P. W. Brophy, Illinois

*Judicial Council*: Drs. S. N. Benham, Pennsylvania; J. M. Toner, Washington, D. C.; D. A. Linthicum, Arkansas; William Brodie, Michigan; H. S. Holton, Vermont; A. B. Sloan, Missouri; R. Beverly Cole, California.

Dr. D. S. Reynolds, of Kentucky, delivered the address of the Chairman of the Section on Ophthalmology, etc., which was appropriately referred. The reports of the Committee on Necrology and secretaries of Sections were also presented. The report of the Section on State Medicine, containing a resolution offered by Dr. Martin, of

Boston, recommending investigation of the vaccine farms and the methods of propagating bovine virus, was adopted, and a committee of three appointed for the purpose, and one hundred dollars appropriated for expenses.

Dr. William H. Beech, of Ohio, chairman, submitted the report of the committee appointed on social position of members of the medical staff of the army and navy, in which he asked that the committee be continued. In doing so, he argued that the principle of promotion which prevails among army officers and in the other departments of the government should be extended to the medical staff.

On motion of Dr. Davis, the matter was laid on the table.

*Resolution of Thanks.*—The Convention passed a resolution of thanks to the Committee of Arrangements of this Association, for the faithful attention they have given to their duties and requirements; to the medical profession and citizens of Richmond, for their hospitality and endeavors to make the meeting pleasant and agreeable; to Drs. McCaw and McGuire for the elegant special entertainment given by them at the Westmoreland Club; to Mr. McClure, superintendent of the Telephone Company, for special facilities given the Committee of Arrangements and the Association; to Vice-President Parsons, of the Richmond & Alleghany Railroad; to Mr. Powell, manager of the Richmond Theatre, and to others who had contributed to the pleasure and comfort of the members.

President Hodgen, in a short speech, then declared the Association adjourned.

